# A retrospective study on disease management in children and adolescents with phenylketonuria during the Covid-19 pandemic lockdown in Austria

**DOI:** 10.1186/s13023-021-01996-x

**Published:** 2021-08-19

**Authors:** Marion Herle, Michaela Brunner-Krainz, Daniela Karall, Bernadette Goeschl, Dorothea Möslinger, Joachim Zobel, Barbara Plecko, Sabine Scholl-Bürgi, Johannes Spenger, Saskia B. Wortmann, Martina Huemer

**Affiliations:** 1 Department of Pediatrics and Adolescent Medicine, Division of Pediatric Pulmonology, Allergology and Endocrinology, Medical University of Vienna, Vienna, Austria; 2 Department of Pediatrics and Adolescent Medicine, Division of General Pediatrics, Medical University of Graz, Graz, Austria; 3 Department of Pediatrics I, Inherited Metabolic Disorders, Medical University of Innsbruck, Innsbruck, Austria; 4 University Children’s Hospital Salzburg, Paracelsus Medical University, Salzburg, Austria; 5 Amalia Children’s Hospital, Radboudumc, Nijmegen, The Netherlands; 6 Division of Metabolism and Children’s Research Center, University Children’s Hospital Zürich, Zürich, Switzerland; 7 Department of Paediatrics, Landeskrankenhaus Bregenz, Carl-Pedenz-Str. 2, 6900 Bregenz, Austria

**Keywords:** Health system resources, Patient self-management, Adherence

## Abstract

**Background:**

In classical phenylketonuria (PKU) phenylalanine (Phe) accumulates due to functional impairment of the enzyme phenylalanine hydroxylase caused by pathogenic variants in the *PAH* gene. PKU treatment prevents severe cognitive impairment. Blood Phe concentration is the main biochemical monitoring parameter. Between appointments and venous blood sampling, Austrian PKU patients send dried blood spots (DBS) for Phe measurements to their centre. Coronavirus disease-19 (COVID-19), caused by the SARS CoV-2 virus, was classified as a pandemic by the World Health Organization in March 2020. In Austria, two nationwide lockdowns were installed during the first and second pandemic wave with variable regional and national restrictions in between. This retrospective questionnaire study compared the frequency of Phe measurements and Phe concentrations during lockdown with the respective period of the previous year in children and adolescents with PKU and explored potential influencing factors.

**Results:**

77 patients (30 female, 47 male; mean age 12.4 [8–19] years in 2020) from five centres were included. The decline of venous samples taken on appointments in 2020 did not reach significance but the number of patients with none or only one DBS tripled from 4 (5.2%) in 2019 to 12 (15.6%) in 2020. Significantly more patients had a decline than a rise in the number of DBS sent in between 2019 and 2020 (*p* < 0.001; Chi^2^ = 14.79). Especially patients ≥ 16 years sent significantly less DBS in 2020 (T = 156, *p* = 0.02, r = 0.49). In patients who adhered to DBS measurements, Phe concentrations remained stable. Male or female sex and dietary only versus dietary plus sapropterin treatment did not influence frequency of measurements and median Phe.

**Conclusion:**

During the COVID pandemic, the number of PKU patients who stopped sending DBS to their metabolic centre increased significantly, especially among those older than 16 years. Those who kept up sending DBS maintained stable Phe concentrations. Our follow-up system, which is based on DBS sent in by patients to trigger communication with the metabolic team served adherent patients well. It failed, however, to actively retrieve patients who stopped or reduced Phe measurements.

## Background

Phenylketonuria (PKU) is an inborn error of phenylalanine (Phe) metabolism in which the enzyme phenylalanine hydroxylase that metabolises Phe to tyrosine is functionally impaired due to variants in the *PAH* gene [1, 2]. Inheritance is autosomal recessive. PKU is a rare disease with an incidence of about 1:10.000 [1, 2]. Untreated, classical PKU leads to severe, early cognitive impairment caused by Phe neurotoxicity. PKU is an ideal disease for newborn screening as cognitive decline is prevented by treatment. Phe concentration is the main biochemical monitoring parameter [1–3].

Mainstay of treatment is a natural protein-restricted diet mostly complemented with special Phe-free amino acid supplements. The oral drug sapropterin can lower Phe in patients with tetrahydrobiopterin (BH4) responsive variants. The recently introduced subcutaneously applied enzyme replacement therapy for PKU, pegvaliase, is licensed for patients from 16 years [3].

In Austria, most patients with PKU are followed in five metabolic centres. Patients come for appointments including venous blood sampling for a complete profile of amino acids and other parameters one to four times per year, depending on age and metabolic stability. Between scheduled visits, patients send dried blood spots (DBS) for Phe (and tyrosine) measurements to the centre. If Phe exceeds the recommended range, the metabolic team actively contacts parents and patients by phone for counselling.

Coronavirus disease-19 (COVID-19), caused by the SARS CoV-2 virus, was classified as a pandemic by the World Health Organization in March 2020. The first Austrian cases of SARS CoV-2 virus infections became evident on February 25th 2020 and the first deaths from COVID-19 occurred in March 2020. A nationwide lockdown including school closures was installed from March 16th to the 4th of May 2020, followed by partial re-opening until the summer holidays in July and August. During the second wave of the pandemic, strict regulations from September 2020 were followed by a second lockdown phase with school closures from November 17th to December 7th 2020 [4].

Although children and adolescents are at less risk for severe courses of COVID-19 disease, they may suffer from indirect effects of the pandemic such as impaired access to chronic and acute care, obesogenic and generally more unhealthy eating and behavioural habits associated with the altered everyday life due to pandemic-associated restrictions and regulations as well as psychological burdens [4–6]. The indirect impact of the pandemic proved even worse for children with chronic health conditions [6]. Globally, inborn metabolic disease-related services were reduced to 60–80% of normal during 3 months of lockdown in 2020 compared to the same period in 2019 indicating a profound impact of the restrictions on patient management and care [7]. For PKU, it is unknown whether impaired management and care applies to all affected children, or mainly to socially deprived individuals [8] or to those with pre-existing psychological burdens [9].

This retrospective study aimed to investigate whether the number of Phe measurements and Phe concentrations differed between times of lockdown compared to the same period of the previous year 2019 and whether age, sex, metabolic control and type of treatment influence changes in number of measurements and Phe concentrations.

## Methods

This retrospective survey was restricted to children with classical PKU who were in 2019 already attending school and who would thus be most affected in their everyday life by the lockdown measures. Metabolic teams of five metabolic centres in Austria in which approximately 90% of the country’s paediatric PKU patients are followed answered survey questions on sex, year of birth and treatment (diet, sapropterin, and enzyme replacement), number of Phe measurements, Phe concentrations from DBS sent from home, number of regular metabolic clinic visits and Phe concentrations from venous samples taken on these occasions, and the number of telemedicine consultations in their patients with classical PKU. Reporting periods for Phe measurements were from 16/3/2020 to 4/12/2020 and from 16/3/2019 to 4/12/2019 (comparison period). Measurement intervals were interpreted according to the age-adjusted control frequency for Phe and Phe target concentrations as recommended by the centres.

### Statistical analysis

Age, sex, type of treatment, number of Phe-measurements, number of regular metabolic clinic visits, number of telemedicine consultations and average Phe concentrations (from DBS and venous samples) for the two measurement periods were analysed descriptively. If patients had more than one Phe measurement from venous samples, the mean was calculated; for Phe measured from DBS, the median (Mdn) was used. The number of patients with a median Phe concentration (from DBS) within the recommended range during one or both measurement periods was determined and confidence intervals (95% CI by modified Wald method) were computed.

Since average Phe concentrations and number of Phe-measurements were not normally distributed between patients, Wilcoxon signed-rank test was applied to compare these variables between the two measurement periods. Analyses were repeated for subgroups of patients (age groups: 8–10/10–12/12–16/ > 16 years; sex: male/female; treatment: with/without sapropterin, average Phe from DBS in 2019: within/above recommended range). For significant results, effect sizes (r) are reported. By convention, effect sizes > 0.2 are considered small effects, > 0.5 medium effects and > 0.8 large effects [10].

For categorical data (0 vs. 1–2 venous samples; average Phe from DBS within versus above the recommended range) Fisher’s exact test was calculated. Calculations were done with SPSS (IBM SPSS Statistics 25.0) and Graphpad [11]. *P* values (two-tailed) ≤ 0.05 were considered statistically significant, for multiple comparisons, Bonferroni–Holm-correction was applied.

## Results

Data from 77 school-age children and adolescents (30 female, 47 male; mean age 12.4 (8–19) years) followed in five metabolic centres in Austria were included. Twenty (26%) were between 8 and 10 years, 18 (23.4%) between 10 and 12 years, 19 (24.7%) between 12 and 16 and 20 (26%) were between 16 and 19 years old. All were on dietary treatment and 11 (14.3%) were additionally treated with sapropterin.

Most centres recommended monthly Phe-measurements for children between 6 and 16 years, but some centres extended intervals in very stable patients. Recommendations to patients older than 16 years were less homogeneous with intervals from monthly to every 6 months. Interestingly, recommendations for Phe target concentrations differed between centres. All except two recommendations followed at least one of the available guidelines or recommendations [12–16]; 13 of 20 recommendations followed the European guidelines [14].

**Fig. 1:**
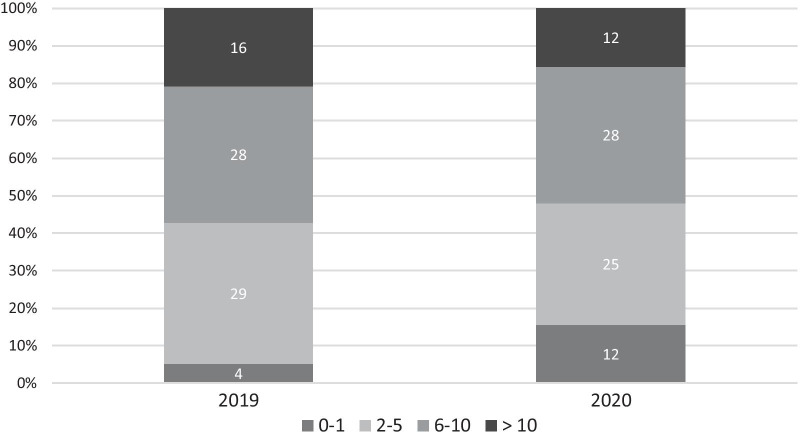
Phe measurements from DBS in 2019 and 2020

The average number of Phe measurements from DBS (Mdn = 7 in 2019 and 6 in 2020) differed significantly between the two measurement periods (*T* = 1602, *p* < 0.001, *r* = 0.28). Overall, the number of patients with none or only one DBS increased from 4 (5.2%) in 2019 to 12 (15.6%) in 2020. Ten patients had not sent a single DBS to the laboratory in 2020. Significantly more patients had a decline than a rise in their number of DBS sent in for Phe measurements from 2019 to 2020 (*p* < 0.001; Chi^2^ = 14.79) (Fig. 1).

Subgroup analysis according to age groups revealed that patients ≥ 16 years sent significantly less DBS in 2020 (*T* = 156, *p* = 0.02, *r* = 0.49).

**Fig. 2:**
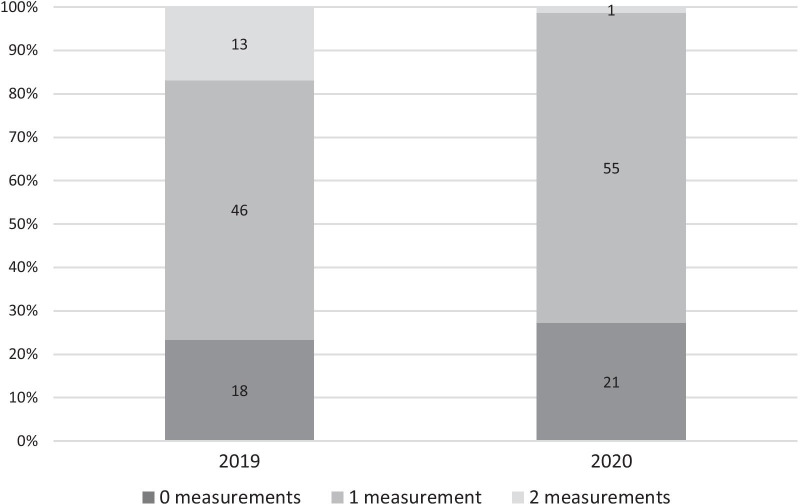
Measurements from venous blood samples in 2019 and 2020

Measurements from venous blood samples are exclusively taken at appointments to the outpatients’ clinic. Whereas 13 patients (16.9%) had two appointments in 2019, only one patient (1.3%) had two regular check-ups in 2020 (Fig. 2). Appointments to the outpatients’ clinic were not generally replaced by telemedicine consultations; only four patients (5.2%) received such a consultation. The number of patients without any appointment and venous blood sampling did not differ significantly between the two measurement periods (n = 21, 27.3% in 2020, n = 18, 23.4% in 2019; *p* = 0.71).

Median Phe concentrations from DBS sent from home (n = 67 patients; 424 µmol/l in both years) and median Phe concentrations from venous blood samples (n = 48 patients; 472 µmol/l in 2019 and 478 µmol/l in 2020;) did not differ significantly between 2019 and 2020 (Phe from DBS: *T* = 782.5, *p* = 1.00; Phe from venous blood samples: *T* = 602.5, *p* = 0.88).

**Fig. 3:**
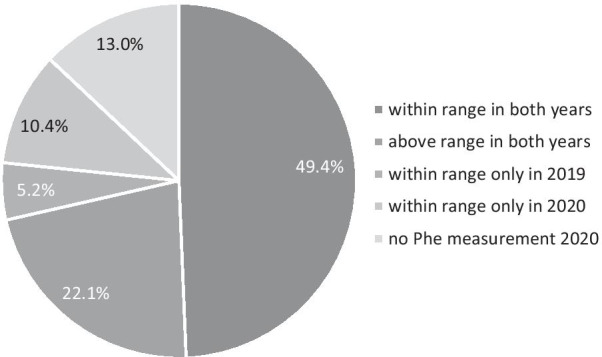
Metabolic control in 2019 and 2020

Forty-five patients (58.4%, 95% CI 47.3–68.8%) managed to keep their median Phe concentrations within the range recommended by their centre in 2019, 46 (59.7%, 95% CI 48.6–70.0%) in 2020 (difference not significant, *p* = 0.23), and 38 (49.4%, 95% CI 38.5–60.3%) patients in both years (Fig. 3).

Patients with a median Phe concentration within the recommended range in 2019 sent significantly less DBS in for Phe measurement (Mdn 2019 = 7; Mdn 2020 = 5; *T* = 577, *p* = 0.01, *r* = 0.36). Descriptively, they had higher Phe values from DBS in 2020 than in 2019, but this difference was not significant (Mdn 272 µmol/l in 2019 versus 375 µmol/l = in 2020, *T* = 592, *p* = 0.15, r = 0.27).

In patients with a median Phe above the recommended range in 2019 the number of Phe measurements as well as the average Phe concentrations did not differ significantly between the two measurement periods (Phe-measurements: *T* = 264, *p* = 0.49; Phe concentrations: *T* = 156.5*, p* = 1.00).

Further subgroup analyses for male or female sex, and dietary only versus dietary plus sapropterin treatment revealed no association with changes in frequency of DBS-measurements and median Phe between 2019 and 2020.

## Discussion

Our retrospective analysis focused on Phe concentrations, number of measurements of Phe from DBS, and number of clinical appointments including Phe assessment from venous blood samples in the year of the pandemic compared to the year before. During the pandemic, patients had fewer appointments and, consecutively, less venous blood samples taken. This is in line with the observation that individuals with a chronic illness experienced significantly more cancellations of scheduled medical procedures and appointments [8] and that both paediatric routine [17﻿] and specific [18] care declined during the lockdown.

Even more prominent, though, was the effect on the low-threshold measurement of Phe from DBS. Sending DBS from home requires no further resources than a post office (which were open during lockdown) or a letterbox. Nevertheless, adherence to the recommended measurement intervals decreased significantly in the pandemic period. Especially adolescents over 16 years sent significantly less DBS in and the number of patients who had sent one or no sample at all for analysis tripled from 5.2 to 15.6%. These findings suggest that during the lockdown a proportion of our patients, especially older teenagers, lost connection to the metabolic centre.

Adherence to treatment in PKU in terms of Phe concentrations in the target range generally declines with age [19]. The frequency of Phe measurement has not been a main target of such analyses. While most children up to 12 years have average Phe in the recommended range, only 49% of teenagers reach such good metabolic control [20, 21]. Teens are easily lost to follow-up and may be lost forever in this vulnerable period, as the low numbers of young adults (41%) and patients > 30 years old (31%) with Phe in the target range suggest [20, 21]. Especially for teens but probably for the majority of paediatric PKU patients an active, communication-seeking follow-up e.g. by low-threshold reminders sent by digital messaging services or by e-mail [18﻿] as well as structured telemedicine appointments [22–24] would most probably have been helpful to improve adherence and patient satisfaction [22–25].

Telemedicine as a substitute for outpatient clinic appointment had only been established in a single centre in Austria, which offered this service to only four PKU patients; its effects could not be quantified. Telemedicine has only slowly been introduced into the health system. In other settings e.g. in Turkey [22] or in Italy [24] it proved to be an effective tool during the pandemic. The authors reported more Phe concentrations in the target ranges [22, 24]. Main characteristic of the systems employed here were timely measurement of and comment on Phe values from DBS with recommendations on how to proceed further [22] and a complete and effective switch from in-person to remote consultations [24]. In Austria, a generally similar system with short Phe measurement turnaround times is the established way of care for PKU patients. Phe results from DBS sent to the centre as well as any actions to be taken are communicated to the patient/family by phone, usually by an experienced member of the metabolic team (e.g. a dietician). This system remained unaffected by the pandemic situation, which may explain that patients who continued to adhere to the recommended DBS measurement intervals had stable Phe concentrations during the pandemic. Since the incoming DBS starts the process of care, this system failed for patients who stopped sending them.

Male or female sex of the patient or type of treatment (diet, medication) did not influence the number of measurements or Phe concentrations during the pandemic. Interestingly, patients with good Phe control in 2019 sent significantly less DBS for analysis and tended to have higher Phe values in 2020 (yet, median values were still within the target range). We hypothesize that patients who had their disease management actions well embedded in their daily routine may have partially lost this control when everyday life changed so significantly during the pandemic. Beyond the mere change of everyday life, diminished adherence—especially of adolescents—may also be caused by the increased stress and emotional disturbances children and adolescents experienced during the pandemic with lockdowns and school closures. During the pandemic, adolescents reported not only more worries and fears but also feelings of overtiredness, underactivity, withdrawal and sadness [26] which make active disease management in terms of sending samples less probable.

## Conclusion

During the COVID-pandemic, PKU patients had less appointments and venous blood sampling for complete amino acid assessment. Some PKU patients stopped sending DBS to their metabolic centre. Those who kept up their follow-up maintained stable Phe values. We conclude that our follow-up system, which is based on DBS sent in by patients/families to trigger communication with the metabolic team, was sufficient in itself and served adherent patients well. It failed, however, to actively retrieve patients who stopped or reduced DBS measurements. Telemedicine and active contacting of patients should complement systems of care for PKU and other metabolic patients not only during but also after the pandemic.

## Data Availability

Please contact author for data requests.
